# Impact of Esterification with Octenyl Succinic Anhydride on the Structural Characteristics and Glucose Response in Mice of Wheat Starch

**DOI:** 10.3390/foods13091395

**Published:** 2024-05-01

**Authors:** Hyun Sung Lee, Gyeong A Jeong, Seokwon Lim, Chang Joo Lee

**Affiliations:** 1 Enterprise Solution Research Center, Korea Food Research Institute, Wanju 55365, Jeollabuk-do, Republic of Korea; lhs0510@kfri.re.kr; 2 Department of Food Science and Biotechnology, Wonkwang University, Iksan 54538, Jeollabuk-do, Republic of Korea; jka0719@naver.com; 3 Department of Food Science and Biotechnology, Gachon University, Seongnam-si 13120, Gyeonggi-do, Republic of Korea; slim@gachon.ac.kr

**Keywords:** octenyl succinic anhydride, esterification, resistant starch, glucose response, low calorie

## Abstract

In this study, we investigated the structural properties and digestibility of wheat starch treated with octenyl succinic anhydride (OSA). For the experiment, the samples were reacted with 2, 4, 6, 8, and 10% OSA (pH 8.5–9.0) for 2 h. A light micrograph showed that there was no difference in the morphology and Maltese cross between native and OSA-treated starch. The X-ray diffraction (XRD) patterns of the native and OSA-treated starches showed typical A-type diffraction. In addition, the Fourier transform infrared (FT-IR) spectrum showed a distinct carbonyl peak at approximately 1730 cm^−1^, indicating the stretching vibration of the C=O bond of the ester group. The degree of substitution (DS) and content of resistant starch (RS) increased with increasing concentrations of treated OSA because of the increase in ester bonds. In particular, RS was thermostable compared to the RS content in uncooked and cooked starch. Blood glucose levels and response in vivo decreased as the OSA concentration increased. Treatment of wheat starch with 8% OSA concentration produced 35.6% heat-stable resistant starch. These results suggest that starch modified with OSA can be used to produce functional foods for diabetes.

## 1. Introduction

Starch is a major energy source, and many crops grown for human consumption worldwide, including wheat, rice, corn, cassava, and potatoes, are sources of starch. In particular, wheat has become the most widely produced grain and staple food in recent years, and wheat-based foods account for approximately 20% of the global energy intake [1,2]. Natural starches, including wheat starches, have insufficient process tolerance and functional limitations, rendering them unsuitable for most industrial food uses [3]. Therefore, many studies have been conducted to improve the properties of starch to maintain its appearance and texture during food processing and expand the scope of its application [4,5,6,7,8]. To accomplish the above goals, among the modifications of starch, chemical modification involves the esterification of the available hydroxyl groups in glucose monomers.

Starch esterification using octenyl succinic anhydride (OSA) induces starch denaturation by partially substituting a hydroxyl group with a hydrophobic substituent. In general, starch modified with OSA is synthesized through esterification with OSA in an aqueous slurry under weakly basic conditions, and several studies have reported modified starch being obtained using this method [9,10,11,12]. OSA starch has the characteristic of increasing slowly digestible starch and resistant starch fraction [13]. The starch fraction of OSA starch has health benefits and is of increasing application and interest in the food industry [14]. However, research on the relationship between the starch molecular structure and the digestibility of OSA starch is still lacking [13,15]. Furthermore, starch modified with OSA can be applied to food within the parameters determined by the US Food and Drug Administration, with a permitted usage of up to 3% starch weight ratio and a degree of substitution (DS) not exceeding 0.02 [16].

In general, starch is nutritionally divided into rapidly digestible starch (RDS), slowly digestible starch (SDS), and resistant starch (RS). Unlike RDS and SDS, which are fully digested and absorbed in the small intestine, RS is mostly undigested and fermented in the large intestine by intestinal microflora [17]. Resistant starch is beneficial to the human body as it is fermented into short-chain fatty acids and metabolites by microorganisms in the large intestine. In addition, resistant starch has various physiological functions, such as preventing type 2 diabetes and intestinal inflammation due to its ability to stabilize blood sugar levels after meals [18]. Therefore, among the properties of starch modified by OSA treatment, an increase in the RS content provides clues of materials necessary to develop new low-calorie foods [19]. Recently, excessive calorie intake due to changes in eating habits caused by modern industrial development has led to an increase in the prevalence of obesity, arteriosclerosis, and heart disease. This has increased the desire for low-calorie materials, including RS [20]. Therefore, this study aimed to investigate the possibility of developing a new food material by examining the structural characteristics and digestibility in vivo in the modified wheat starch according to the OSA concentration.

## 2. Materials and Methods

Wheat starch was purchased from Roquette Frères (Lestrem, France), and OSA was purchased from Sigma–Aldrich (2-Octen-1-ylsuccinic anhydride; 416487; Sigma–Aldrich; St. Louis, MO, USA). The enzymes porcine pancreatin (P7545; activity, 8× United States Pharmacopeia [USP]/g; Sigma–Aldrich; St. Louis, MO, USA) and amyloglucosidase (AMG 300 L; activity, 300 amyloglucosidase activity [AGU]/mL; Novozymes Inc.; Bagsvaerd, Denmark) were utilized to digest the starch. All other chemicals and reagents used in this study were of analytical grade.

### 2.1. Preparation of OSA-Treated Starch

OSA-treated wheat starch was prepared as described by Zhang et al. [21] with minor modifications. Distilled water was added to 25 g wheat starch (Roquette Frères, Lestrem, France) to prepare a 35% starch slurry. The starch slurry was temperature-equilibrated in a 35 °C water bath for 15 min, and the pH was adjusted to 8.5–9.0 using 1 M NaOH. 2-Octen-1-ylsuccinic anhydride (OSA, 416487, Sigma–Aldrich; St. Louis, MO, USA), treated at different starch concentrations (2, 4, 6, 8, and 10%), and reacted with agitation for 2 h. The pH was maintained within the range of 8.5–9.0 using a pH meter (Thermo Orion Star A215, Tewksbury, MA, USA). To terminate the reaction, it was neutralized to a pH of 6.5 using 1 M HCl. Thereafter, the mixture was washed thoroughly with distilled water to remove unreacted OSA and then washed with 95% ethanol. Afterward, the OSA-treated starch was dried in the 40 °C air-drying oven and ground. A control sample of starch was prepared following the same procedure but without OSA treatment.

### 2.2. Light Microscopy

Starch granules were observed under a light microscope (Olympus BX40; Olympus Optical Co., Ltd., Tokyo, Japan) with and without a polarizing plate. Glycerol was used to disperse all the samples on a glass slide to reduce air bubbles. Starch granule size was measured via image analysis using ToupView version 3.7.7158 for Windows (ToupTek Photonics Co., Ltd., Hangzhou, China).

### 2.3. Fourier-Transform–Infrared (FT-IR) Spectroscopy

IR spectra were obtained using an FT-IR spectrometer (Spectrum TWO, Perkin–Elmer, Shelton, CT, USA). The spectra were measured in the range of 4000–400 cm^−1^ in the transmission mode at a resolution of 4 cm^−1^ and were normalized to the 1315 cm^−1^ peak of the CH_2_ vibration of the starch. The samples were diluted using KBr (1:100, *v*/*v*) before analysis.

### 2.4. Determination of the Degree of Substitution (DS)

The DS was determined to estimate the average number of hydroxyl groups substituted by the OSA hydroglucose unit in the starch. The measurements were performed according to the method described by Zhang et al. [21] with some modifications. An OSA-treated starch (0.5 g, dry weight) was accurately weighed and dispersed in 2.5 mL of 2.5 M HCl isopropyl alcohol solution by stirring for 30 min. Subsequently, 10 mL of 90% (*v*/*v*) aqueous isopropyl alcohol solution was added, and the slurry was stirred for an additional 10 min. The suspension was filtered through a glass filter, and the residue was washed with a 90% isopropyl alcohol solution until no Cl^−^ could be detected (using 0.1 M AgNO_3_ solution). Starch was redispersed in 30 mL of distilled water, and the dispersion was cooked in a boiling water bath for 20 min. The starch solution was titrated with 0.1 M NaOH solution using a pH meter (Orion Star A211, Thermo Scientific, Waltham, MA, USA). Native starch was used as a blank and was titrated simultaneously. DS was calculated using the following Equation (1):

DS = (0.162 × (*A* × *M*)/*W*)/(1 − (0.210 × (*A* × *M*)/*W*)) (1)

where *W* is the sample weight (g), *A* is the volume of NaOH (mL), *M* is the molarity of NaOH, 0.162 is the molecular weight of an anhydrous glucose unit (AGU) of starch, and 0.210 is the molecular weight of OSA.

### 2.5. Determination of In Vitro Digestibility

The in vitro digestibility was measured according to the method described by Englyst et al. [22] with minor modifications, as reported by Na et al. [23]. To prepare the enzyme solutions, porcine pancreatin (2 g) was added to distilled water (24 mL) in a glass beaker (50 mL) and stirred for 10 min. Thereafter, the solution was centrifuged at 1500× *g* for 10 min at 4 °C to obtain the cloudy supernatant. The supernatant (20 mL) was mixed with 0.4 and 3.6 mL of amyloglucosidase and distilled water, respectively. To a microtube (2 mL) containing 30 mg of the starch sample, 0.75 mL of a sodium acetate buffer (0.1 M, pH 5.2) and a glass bead were added and either cooked for 30 min or entirely uncooked. After cooling the tube to 37 °C, 0.75 mL of the enzyme solution was added and incubated in a shaking incubator (240 rpm). The tubes were removed after 20 and 240 min, boiled for 10 min on a heating block to stop the reaction, and cooled at a temperature between 20 and 23 °C. Next, the tubes were centrifuged at 5000× *g* for 10 min at 4 °C. The glucose content of the supernatant was measured using a glucose oxidase-peroxidase kit (Embiel Co., Gunpo, Republic of Korea). The amount of glucose obtained after 20 min of the enzyme reaction at 37 °C corresponded to RDS, and that obtained after incubation for 20–240 min corresponded to SDS. RS was not hydrolyzed after 240 min of incubation.

### 2.6. XRD

The XRD patterns of the starches were investigated using an X-ray diffractometer (MiniFlex-600, Rigaku, Tokyo, Japan), which was operated at 40 kV and 15 mA to produce CuKα radiation of 1.54 Å and scanned through the 2*θ* range of 3°–30° with a 0.02° step size. The relative crystallinity of the starch was calculated using peak-fitting software (Origin version 7.5, OriginLab, Northampton, MA, USA) using the following Equation (2) [23]:

Relative crystallinity (%) = *A_c_*/(*A_a_* + *A_c_*) × 100 (2)

where *A_a_* is the area of the amorphous region, and *A_c_* is the area of the crystalline region.

### 2.7. Gelatinization Parameters

Differential scanning calorimetry (DSC) was performed using a calorimeter (DSC 4000, Perkin–Elmer, Waltham, MA, USA) calibrated with an indium standard to analyze the gelatinization parameters. Distilled water (40 µL) was added to the sample (10 mg) in a stainless-steel pan, which was sealed afterward, weighed again, and incubated for 4 h at 23–25 °C to maintain an even distribution of distilled water. Heat was simultaneously applied to the pans containing the sample and blank from 30 to 130 °C at a rate of 5 °C/min; the blank pan was used as a reference. The onset (*T_o_*), peak (*T_p_*), and conclusion (*T_c_*) temperatures, as well as the gelatinization enthalpy (∆*H*), were measured using the Pyris software (Perkin–Elmer, version 13.3.1.0014) availed by the manufacturer of the differential scanning calorimeter.

### 2.8. Glucose Responses in Mice

Fifty female 4-week-old mice (weighing 20–22 g) from the Institute of Cancer Research were individually housed in an approved laboratory animal facility for a 7-day acclimatization period and then randomly divided into groups of five each for the experiment. Feed and water were supplied freely, the laboratory animal facility was maintained at a temperature of 23 ± 3 °C and humidity of 60 ± 10%, and light and dark were adjusted at 12-h intervals. The mice were fasted for 12 h and then administered a 0.5 mL sample of suspension (7.5%, *w*/*v*) or glucose (7.5%, *w*/*v*) using an oral Zonde needle [24]. Blood samples were collected from the tail vein of each mouse at 0, 30, 60, 90, 120, 150, 180, and 240 min. Serum glucose levels were measured using an Accu-Chek Performa (Roche, Basel, Switzerland). The glucose response was calculated based on the procedure described by Lee et al. [5,25] by comparing the area under the blood glucose response curve of each sample as a percentage of the response to glucose using Origin software (version 7.5; OriginLab, MA, USA).

### 2.9. Statistical Analysis

The experiments were performed in triplicate, and the average values and standard deviations were calculated. Variance analysis was performed using Duncan’s multiple-range test to assess significant differences. Statistical Package for Social Sciences (SPSS) software (version 22.0; IBM, Armonk, NY, USA) was used for statistical analyses.

## 3. Results and Discussion

### 3.1. Morphology of OSA-Treated Starch

**Figure 1:**
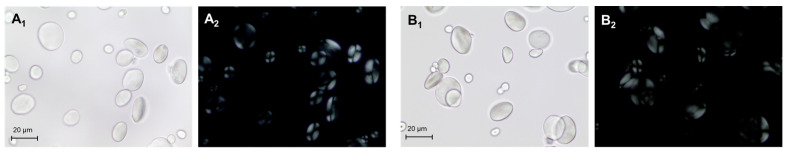

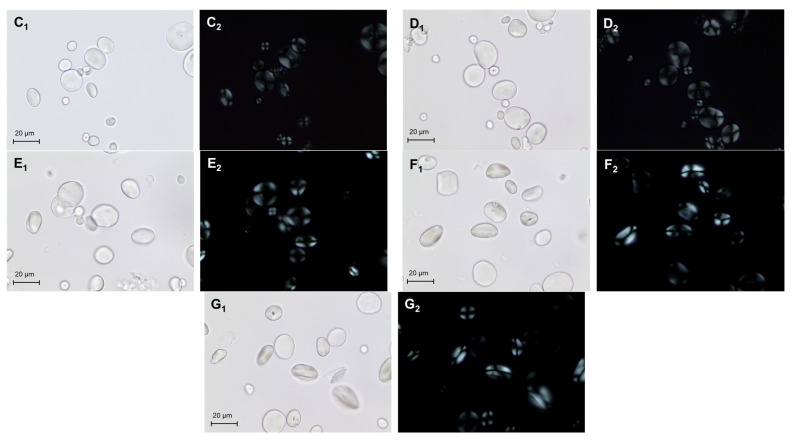
Light micrographs of granules of native and OSA wheat starches: (**A**) native starch; (**B**) control; (**C**) OSA-2%; (**D**) OSA-4%; (**E**) OSA-6%; (**F**) OSA-8%; (**G**) OSA-10%; (**1**) light micrographs and (**2**) under polarized light.

The granular shapes of native and OSA-treated starch samples were observed using an optical microscope (Figure 1). Most granules were spherical or elliptical in shape and similar in size. In addition, the shapes of the starch granules did not change even when the starch was treated with 10% OSA, which was the highest concentration tested in this experiment. Therefore, no morphological differences were observed between the native and OSA-treated starch samples. In addition, the Maltese cross, confirmed under all conditions, shows that the internal structure of the starch maintained a regular arrangement even after OSA treatment. The lack of change in the shape of starch granules indicated that esterification with OSA did not affect the original crystal shape of the starch granules.

### 3.2. DS

The degree of substitution (DS) of the OSA-treated starches is presented in Table 1. Native starch and the control showed significantly lower DS values of 0.001 and 0.006, respectively (*p* < 0.05), and DS increased from 0.044 to 0.091 as the concentration of treated OSA increased. This was probably due to the high reactivity between the starch and OSA molecules under the reaction conditions of this experiment. However, there was no difference in the DS of the 8% and 10% OSA-treated starches. The modification of starch using OSA treatment mainly occurs in the hydroxyl groups of the amorphous part of the starch [26,27]. In addition, OSA is found three to four times more outside than inside the modified starch because some parts of OSA are able to react with the hydroxyl group inside because of the structure of the starch granule [28]. Therefore, the lack of a difference in the DS of 8% and 10% OSA-treated starch was considered to be due to differences in the structure of the starch granules.

### 3.3. FT-IR

**Figure 2:**
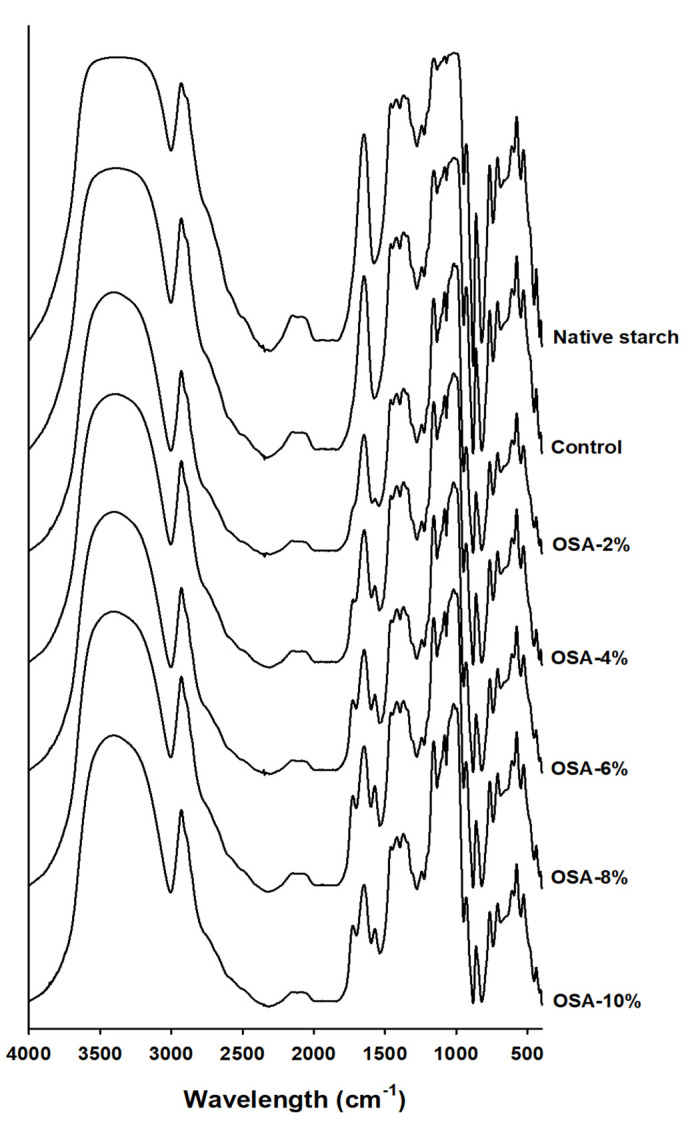
Fourier−transform infrared spectroscopy (FT−IR) of native and OSA wheat starch.

Fourier-transform infrared (FT-IR) spectroscopy was used to determine the functional groups of OSA-treated starch and its structural properties. The peak(s) between 3000 and 3500 cm^−1^ indicated the presence of a hydroxyl group in starch. The peak(s) between 3440 and 2930 cm^−1^ may be related to the stretching vibrations caused by the OH and C-H groups, and the peak(s) at approximately 1730 cm^−1^ corresponds to the stretching of ester carbonyl groups [12,29]. In this study, the peak(s) between 3000 and 3500 cm^−1^ did not show a significant difference between native starch and OSA-treated starch. However, the peak(s) around 1730 cm^−1^, not found in the FT-IR spectrum of natural starch and the control, was confirmed in the OSA-treated starch (Figure 2). In particular, the peak intensity gradually increased as the treated OSA concentration increased, indicating a positive correlation between DS and peak intensity. These results show that OSA successfully reacted with native starch to form an ester bond.

### 3.4. In Vitro Digestibility

The RS, RDS, and SDS contents of the OSA-treated starches are listed in Table 2. The RDS of the uncooked starch sample did not show a trend according to the OSA concentration. However, when the concentration of OSA was increased from 2% to 8%, SDS decreased from 29.0% to 13.9%, whereas RS showed an increasing tendency (23.4%, 27.0%, 31.8%, 35.6%). The cooked starch sample showed the conversion of SDS to RDS, and unlike the uncooked starch sample, the RDS decreased (76.3% to 66.4%) as the concentration of treated OSA increased (2% to 8%). Nevertheless, RS showed a positive correlation with the OSA-treated concentration, similar to the uncooked starch sample. It is considered that SDS or RDS were converted to RS owing to increased crosslinking by esterification due to the OSA treatment. In addition, when treated with the same OSA, the RS of the uncooked and cooked starch samples showed little difference, indicating that the RS was thermostable. However, there was no significant difference in the RS of starch samples treated with 8% and 10% OSA. This result was similar to the lack of significant difference in DS under the same conditions (*p* > 0.05). These results are generally consistent with a previous report that alternative starch has a high resistance to diffusion, and this resistance is directly proportional to DS [30].

### 3.5. XRD

**Figure 3:**
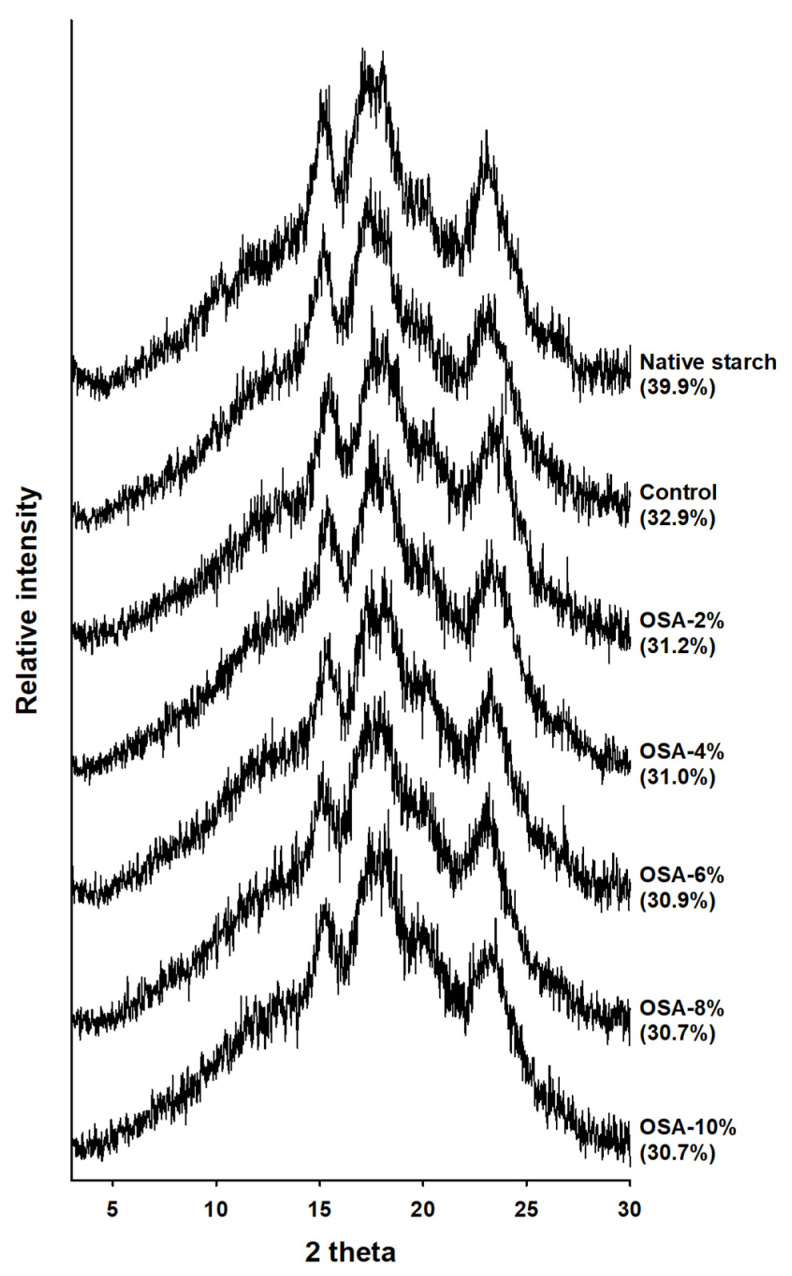
XRD patterns of native and OSA wheat starches. The numbers in parentheses indicate the percentages of crystallinity.

X-ray diffraction has been widely used to elucidate the crystal structure of starch granules [31]. The internal order of the starch granules was demonstrated by A-, B-, and C-type diffraction patterns [31,32]. In this study, The XRD patterns of both the native and OSA-treated wheat starch exhibited peaks at 15°, 17°, 18°, and 23° (2 *θ*), which is a typical A-type diffraction (Figure 3). Therefore, the OSA treatment did not affect starch diffraction. However, as the OSA processing concentration increased, the degree of crystallinity decreased from 31.2% to 30.7% compared with that of native starch at 39.9%. Although the XRD patterns were retained, the degree of crystallinity decreased. In addition, DS increased in an aqueous medium under alkaline conditions, depending on the OSA concentration (Table 1). The modified starch maintained its crystal pattern and showed little change in the crystallization rates of starch granules (Figure 3). These results suggest that OSA substitution occurs mainly in the amorphous region of starch, which is consistent with previous studies [12,28,33,34]. Therefore, in samples treated with OSA at different levels, crystallinity was maintained with a typical A-type peak; however, as OSA levels increased, crystallinity was gradually destroyed.

### 3.6. Thermal Properties

The gelatinization parameters of the OSA-treated wheat starch, such as *T_o_*, *T_p_*, *T_c_*, and Δ*H*, were studied, and their thermal properties are presented in Table 3. Native starch was shown as the typical endotherm of wheat, and *T_o_*, *T_p_*, *T_c_*, and Δ*H* for native starch are 59.2, 64.8, 73.4 °C, and 6.75 J/g, respectively. These results agree with *T_o_*, *T_p_*, *T_c_*, and Δ*H* reported in previous studies [35]. In general, the gelatinization temperature (*T_p_*) is related to the crystallinity perfection of starch granules, and the gelatinization enthalpy (Δ*H*) is related to the degree of crystallinity [36]. In this study, the *T_p_* value increased significantly (*p* < 0.05) as the concentration of OSA increased, confirming that the crystallinity perfection was increased by ester bonds and cross-linking bonds due to OSA treatment. In addition, Δ*H* was decreased in inverse proportion to the concentration of OSA treatment. Since Δ*H* was denoted by the energy required to loosen the double helix structure of starch, a low Δ*H* value indicated a relatively large amount of starch with destroyed crystals. Starch is composed of a double-helical structure, and OSA treatment induces the formation of new ester and cross-linking bonds within starch, leading to its development in various forms. Consequently, the unraveling of the double-helical structure increased, resulting in a significant increase in the *T_c_*–*T_o_* value with the increasing OSA treatment concentration. These results are consistent with those of a study by Na et al. [23], who observed that the double helical structure of starch was unraveled by acid and heat treatments, resulting in an increase in the gelatinization temperature range (*T_c_*–*T_o_*).

### 3.7. Glucose Responses in Mice

**Figure 4:**
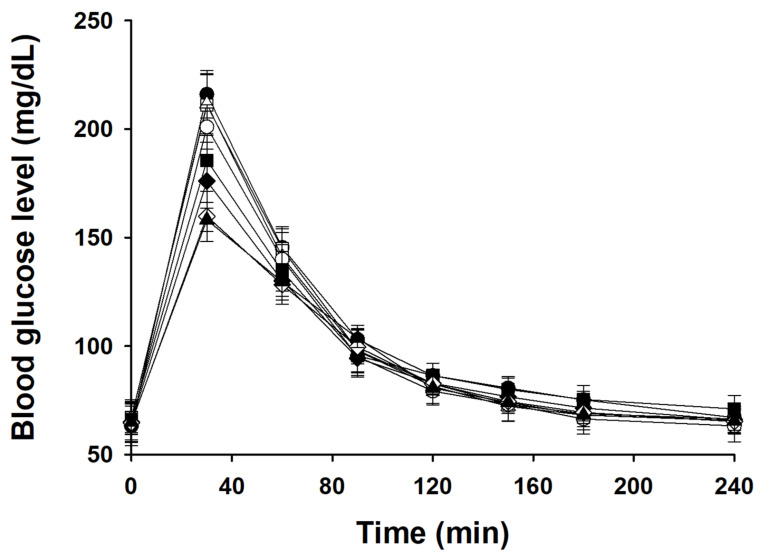
Mean blood glucose concentration in mice after intake of glucose, native starch, control, OSA-2%, OSA-4%, OSA-6%, OSA-8%, and OSA-10%: ●, glucose; □, native starch; △, control; ○, OSA-2%; ■, OSA-4%; ◆, OSA-6%; ◇, OSA-8%, and ▲, OSA-10%.

**Figure 5:**
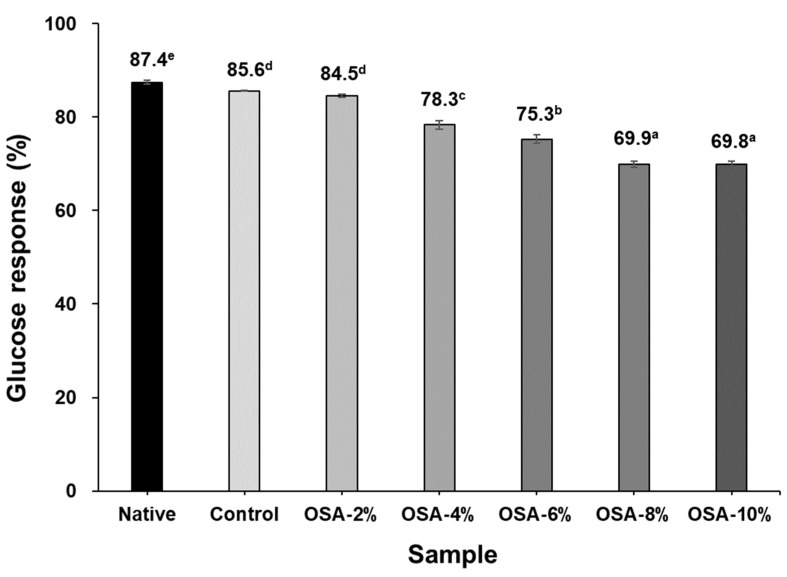
Glucose response of native and OSA wheat starches. Error bars represent standard deviations, and different letters indicate significant differences (*p* < 0.05) between treatments.

Figure 4 shows postprandial glucose concentrations in mice after the uptake of glucose, native samples, and OSA-treated samples. We compared the areas defined by the blood glucose response and glucose baseline in accordance with the concept of glycemic response [37]. The maximum blood glucose levels in all samples were reached at 30 min, following which the levels declined sharply (Figure 4). The peak blood glucose level in the glucose sample was 220 mg/dL. As the concentration of starch in the OSA treatment increased from 2% to 8%, the peak blood glucose level decreased from 200 to 155 mg/dL. It appears that the higher the RS content in starch, the lower the blood glucose level. All samples had glucose response measured at 30-min intervals. The small difference in the measured blood glucose level after 30 min is thought to be due to the difference in the SDS content in starch (Table 2). Moreover, the blood glucose response level in mice indicates the digestibility of starch compared with that of glucose. As the concentration of OSA increased from 2% to 8%, the blood glucose response level decreased from 84.5% to 69.9% (Figure 5), confirming that the higher the degree of starch modification using OSA, the lower the starch digestibility (Table 2). In particular, the higher the ratio of RS in starch, the lower the blood glucose response; therefore, an increase in the RS ratio in modified starch plays a major role in reducing the blood glucose response. These results are consistent with previous reports that ingestion of RS-rich potato starch reduces blood glucose levels and responses in rats [5,6].

## 4. Conclusions

Wheat starch rich in RS was prepared using various concentrations of OSA and used in this study. FT-IR spectroscopy indicated the characteristic absorption of ester carbonyl groups in OSA-treated starch at approximately 1730 cm^−1^ and confirmed that DS increased with an increase in the concentration of treated OSA. The RS content of OSA-treated, cooked, and uncooked starch was higher than that of natural starch; the highest RS content was 35.6%, which was observed for uncooked starch treated with 8% OSA. In addition, for cooked starch treated with 8% OSA, the RS content was 32.7%, confirming the thermal stability of RS. In addition, mouse experiments showed that the ingestion of RS-rich, OSA-treated wheat starch reduced both blood glucose levels and the blood glucose response. This study provides basic information for the development of thermally stable and digestibility-modified starches by treating wheat with OSA. For the preparation of heat-stable resistant starch using OSA, processing at a concentration of 8% OSA is most appropriate. Therefore, the OSA treatment of starch can be applied to food production as a material for reducing calories and preventing diabetes.

## Figures and Tables

**Table 1:** Degree of substitution of native and OSA wheat starches.

Sample	Degree of Substitution
Native starch	0.001 ± 0.001 ^a^
Control	0.006 ± 0.003 ^a^
OSA-2%	0.044 ± 0.007 ^b^
OSA-4%	0.068 ± 0.002 ^c^
OSA-6%	0.079 ± 0.003 ^d^
OSA-8%	0.090 ± 0.002 ^e^
OSA-10%	0.091 ± 0.001 ^e^

^a–e^ The values with different superscripts within a column are significantly different (*p* < 0.05) according to Duncan’s multiple range test.

**Table 2:** Contents of rapidly digestible starch (RDS), slowly digestible starch (SDS), and resistant starch (RS) of uncooked and cooked native and OSA wheat starches.

Sample	Uncooked Starch			Cooked Starch		
	RDS (%)	SDS (%)	RS (%)	RDS (%)	SDS (%)	RS (%)
Native starch	51.0 ± 1.72 ^c^	43.8 ± 3.13 ^f^	5.18 ± 1.73 ^a^	93.1 ± 0.75 ^e^	1.77 ± 0.99 ^abc^	5.16 ± 0.36 ^a^
Control	58.6 ± 1.24 ^d^	35.4 ± 1.23 ^e^	6.08 ± 0.27 ^a^	94.5 ± 1.67 ^e^	1.22 ± 0.28 ^ab^	4.54 ± 1.44 ^a^
OSA-2%	47.6 ± 1.84 ^b^	29.0 ± 1.09 ^d^	23.4 ± 0.99 ^b^	76.3 ± 0.87 ^d^	4.16 ± 0.88 ^d^	19.5 ± 1.68 ^a^
OSA-4%	50.3 ± 0.77 ^c^	22.7 ± 1.01 ^c^	27.0 ± 0.79 ^c^	71.8 ± 0.54 ^c^	2.30 ± 1.00 ^bc^	25.9 ± 0.83 ^b^
OSA-6%	51.4 ± 0.94 ^c^	16.8 ± 1.61 ^b^	31.8 ± 2.10 ^d^	68.7 ± 0.74 ^b^	2.57 ± 0.36 ^c^	28.8 ± 0.38 ^c^
OSA-8%	50.5 ± 1.14 ^c^	13.9 ± 1.15 ^a^	35.6 ± 1.30 ^e^	66.4 ± 0.53 ^a^	0.92 ± 0.58 ^a^	32.7 ± 0.35 ^d^
OSA-10%	44.6 ± 0.94 ^a^	21.4 ± 0.84 ^c^	34.0 ± 0.72 ^e^	66.2 ± 1.58 ^a^	2.01 ± 0.33 ^abc^	31.8 ± 1.60 ^d^

^a–e^ The values with different superscripts within a column are significantly different (*p* < 0.05) according to Duncan’s multiple range test.

**Table 3:** Gelatinization parameters of native and OSA wheat starches.

Sample	*T_o_* (°C)	*T_p_* (°C)	*T_c_* (°C)	*T_c_*–*T_o_* (°C)	Δ*H* (J/g)
Native starch	59.2 ± 0.31 ^a^	64.8 ± 0.13 ^a^	73.4 ± 0.13 ^a^	14.3 ± 0.42 ^a^	6.75 ± 0.46 ^c^
Control	59.2 ± 0.09 ^a^	64.7 ± 0.14 ^a^	73.8 ± 0.25 ^a^	14.6 ± 0.22 ^ab^	6.28 ± 0.18 ^bc^
OSA-2%	59.3 ± 0.20 ^a^	64.8 ± 0.34 ^a^	74.0 ± 0.36 ^a^	14.7 ± 0.53 ^ab^	6.16 ± 0.55 ^bc^
OSA-4%	59.4 ± 0.12 ^a^	64.9 ± 0.12 ^a^	74.3 ± 0.37 ^ab^	14.9 ± 0.43 ^ab^	5.82 ± 0.78 ^abc^
OSA-6%	60.1 ± 0.10 ^b^	65.4 ± 0.15 ^b^	75.1 ± 0.70 ^bc^	15.0 ± 0.78 ^ab^	5.21 ± 0.45 ^ab^
OSA-8%	60.2 ± 0.61 ^b^	65.1 ± 0.10 ^ab^	75.6 ± 0.47 ^c^	15.4 ± 0.41 ^b^	4.82 ± 0.48 ^a^
OSA-10%	59.7 ± 0.11 ^ab^	65.4 ± 0.07 ^b^	75.3 ± 0.21 ^c^	15.6 ± 0.09 ^b^	4.70 ± 0.25 ^a^

*T_o_*, onset temperature; *T_p_*, peak temperature; *T_c_*, conclusion temperature; *T_c_–T_o_*, temperature range of crystal melting; Δ*H*, enthalpy change. ^a–c^ The values with different superscripts within a column are significantly different (*p* < 0.05) according to Duncan’s multiple range test.

## Data Availability

Data will be made available upon reasonable request.

## References

[1] Food and Agriculture Organization (2023). Crop Prospects and Food Situation-Quarterly Global Report 2023.

[2] Bradauskiene V., Vaiciulyte-Funk L., Martinaitiene D., Andruskiene J., Verma A.K., Lima J.P., Serin Y., Catassi C. (2023). Wheat consumption and prevalence of celiac disease: Correlation from a multilevel analysis. Crit. Rev. Food Sci..

[3] Khlestkin V.K., Peltek S.E., Kolchanov N.A. (2018). Review of direct chemical and biochemical transformations of starch. Carbohydr. Polym..

[4] Jinhua H., Jie L., Genyi Z. (2008). Slowly digestible waxy maize starch prepared by octenyl succinic anhydride estrification and heat-moisture treatment: Glycemic response and mechanism. Biomacromolecules.

[5] Lee C.J., Kim Y., Choi S.J., Moon T.W. (2012). Slowly digestible starch from heat-moisture treated waxy potato starch: Preparation, structural characteristics, and glucose response in mice. Food Chem..

[6] Lee C.J., Shin S.I., Kim Y., Choi H.J., Moon T.W. (2011). Structural characteristics and glucose response in mice of potato starch modified by hydrothermal treatments. Carbohydr. Polym..

[7] Na J.H., Kim H.R., Kim Y., Lee J.S., Park H.J., Moon T.W., Lee C.J. (2020). Structure characteristics of low-digestible sweet potato starch prepared by heat-moisture treatment. Int. J. Biol. Macromol..

[8] Ruan H., Chen Q., Fu M., Xu Q., He G. (2009). Preparation and properties of octenyl succinic anhydride modified potato starch. Food Chem..

[9] Bai Y., Shi Y.C. (2011). Structure and preparation of octenyl succinic esters of granular starch, microporous starch and soluble maltodextrin. Carbohydr. Polym..

[10] Bhosale R., Singhal R. (2006). Process optimization for the synthesis of octenyl succinyl derivative of waxy corn and amaranth starches. Carbohydr. Polym..

[11] Liu Z., Li Y., Cui F., Ping L., Song J., Ravee Y., Jin L., Xue Y., Xu J., Li G. (2008). Production of octenyl succinic anhydride-modified waxy corn starch and its characterization. J. Agric. Food Chem..

[12] Altuna L., Herrera M.L., Foresti M.L. (2018). Synthesis and characterization of octenyl succinic anhydride modified starches for food applications. A review of recent literature. Food Hydrocolloid..

[13] Lopez-Silva M., Bello-Perez L.A., Castillo-Rodriguez V.M., Agama-Acevedo E., Alvarez-Ramirez J. (2020). In vitro digestibility characteristics of octenyl succinic acid (OSA) modified starch with different amylose content. Food Chem..

[14] Lehmann U., Robin F. (2007). Slowly digestible starch–its structure and health implications: A review. Trends Food Sci. Technol..

[15] Sweedman M.C., Tizzotti M.J., Schäfer C., Gilbert R.G. (2013). Structure and physicochemical properties of octenyl succinic anhydride modified starches: A review. Carbohydr. Polym..

[16] Miao M., Li R., Jiang B., Cui S.W., Zhang T., Jin Z. (2014). Structure and physicochemical properties of octenyl succinic esters of sugary maize soluble starch and waxy maize starch. Food Chem..

[17] Nugent A. (2005). Health properties of resistant starch. Nutr. Bull..

[18] Jiali L., Wu Z., Liu L., Yang J., Wang L., Li Z., Liu L. (2023). The research advance of resistant starch: Structural characteristics, modification method, immunomodulatory function, and its delivery systems application. Crit. Rev. Food Sci..

[19] Zhang Z., Bao J. (2021). Recent advances in modification approaches, health benefits, and food applications of resistant starch. Starch-Starke.

[20] Lim S.S., Vos T., Flaxman A.D., Danaei G., Shibuya K., Adair-Rohani H., Amann M., Anderson H.R., Andrews K.G., Aryee M. (2012). A comparative risk assessment of burden of disease and injury attributable to 67 risk factors and risk factor clusters in 21 regions, 1990–2010: A systematic analysis for the Global Burden of Disease Study. Lancet.

[21] Zhang B., Mei J., Chen B., Chen H. (2017). Digestibility, physicochemical and structural properties of octenyl succinic anhydride-modified cassava starches with different degree of substitution. Food Chem..

[22] Englyst H.N., Kingman S.M., Cummings H.J. (1992). Classification and measurement of nutritionally important starch fractions. Eur. J. Clin. Nutr..

[23] Na J.H., Jeong G.A., Park H.J., Lee C.J. (2021). Impact of esterification with malic acid on the structural characteristics and in vitro digestibilities of different starches. Int. J. Biol. Macromol..

[24] Mansur A.R., Jeong G.A., Lee C.J. (2022). Preparation, physicochemical properties, and in vivo digestibility of thermostable resistant starch from malic acid-treated wheat starch. Food Res. Int..

[25] Wolever T.M.S. (2004). Effect of blood sampling schedule and method of calculating the area under the curve on validity and precision of glycaemic index values. Br. J. Nutr..

[26] He G.Q., Song X.Y., Ruan H., Chen F. (2006). Octenyl succinic anhydride modified early *indica* rice starches differing in amylose content. J. Agric. Food Chem..

[27] Zainal Abiddin N.F., Yusoff A., Ahmad N. (2018). Effect of octenylsuccinylation on physicochemical, thermal, morphological and stability of octenyl succinic anhydride (OSA) modified sago starch. Food Hydrocolloid..

[28] Shorgen R.L., Vishwanathan A., Felker F., Gross R.A. (2000). Distribution of octenyl succinate groups in octenyl succinic anhydride modified waxy maize starch. Starch-Starke.

[29] Shin S.I., Lee C.J., Kim D.I., Lee H.A., Cheong J.J., Chung J.M., Baik M.Y., Park C.S., Kim C.H., Moon T.W. (2007). Formation, characterization, and glucose response in mice to rice starch with low digestibility produced by citric acid treatment. J. Cereal. Sci..

[30] Viswanathan A. (1999). Effect of degree of substitution of octenyl succinate starch on the emulsification activity on different oil phases. J. Polym. Environ..

[31] Li M.N., Xie Y., Chen H.Q., Zhang B. (2019). Effects of heat-moisture treatment after citric acid esterification on structural properties and digestibility of wheat starch, A-and B-type starch granules. Food Chem..

[32] Shin S.I., Kim H.J., Ha H.J., Lee S.H., Moon T.W. (2005). Effect of hydrothermal treatment on formation and structural characteristics of slowly digestible non-pasted granular sweet potato starch. Starch-Starke.

[33] Zhang B., Tao H., Niu X., Li S., Chen H.Q. (2017). Lysozyme distribution, structural identification, and in vitro release of starch-based microgel-lysozyme complexes. Food Chem..

[34] Zheng Y., Hu L., Ding N., Liu P., Yao C., Zhang H. (2017). Physicochemical and structural characteristics of the octenyl succinic ester of ginkgo starch. Int. J. Biol. Macromol..

[35] Obadi M., Xu B. (2021). Review on the physicochemical properties, modifications, and applications of starches and its common modified forms used in noodle products. Food Hydrocolloid..

[36] Eliasson A.C., Gudmunsson M., Eliasson A.C. (1996). Starch: Physicochemical and functional aspects. Carbohydrates in Foods.

[37] Ma T., Lee C.D. (2021). Effect of high dose resistant starch on human glycemic response. J. Nutr. Med. Diet Care.

